# Post-Translational Modification of Cav1.2 and its Role in Neurodegenerative Diseases

**DOI:** 10.3389/fphar.2021.775087

**Published:** 2022-01-17

**Authors:** Yun Li, Hong Yang, Tianhan He, Liang Zhang, Chao Liu

**Affiliations:** ^1^ Jiangsu Province Key Laboratory of Anesthesiology, Jiangsu Province Key Laboratory of Anesthesia and Analgesia Application Technology, NMPA Key Laboratory for Research and Evaluation of Narcotic and Psychotropic Drugs, School of Anesthesiology, Xuzhou Medical University, Xuzhou, China; ^2^ Department of Neurology, Affiliated Hospital of Qingdao University, Qingdao, China

**Keywords:** Cav1.2, PTM (post-translational modification), neurodegenerative disease, phosphorylation, ubiquitination

## Abstract

Cav1.2 plays an essential role in learning and memory, drug addiction, and neuronal development. Intracellular calcium homeostasis is disrupted in neurodegenerative diseases because of abnormal Cav1.2 channel activity and modification of downstream Ca^2+^ signaling pathways. Multiple post-translational modifications of Cav1.2 have been observed and seem to be closely related to the pathogenesis of neurodegenerative diseases. The specific molecular mechanisms by which Cav1.2 channel activity is regulated remain incompletely understood. Dihydropyridines (DHPs), which are commonly used for hypertension and myocardial ischemia, have been repurposed to treat PD and AD and show protective effects. However, further studies are needed to improve delivery strategies and drug selectivity. Better knowledge of channel modulation and more specific methods for altering Cav1.2 channel function may lead to better therapeutic strategies for neurodegenerative diseases.

## Introduction

Cav1.2, encoded by the *CACNA1C* gene, is a high-voltage-activated (HVA), long-lasting (L-type), and dihydropyridine (DHP)-sensitive calcium channel. Cav1.2 mediates depolarization of the cell membrane potential, calcium (Ca^2+^) influx, and activation of intracellular Ca^2+^ signaling cascades that alter gene expression, protein phosphorylation, and neurotransmitter release. Cellular excitability and signal transduction are affected by factors that modulate Cav1.2 activity. Cav1.2 channels are located in the cardiovascular system, the nervous system, and endocrine glands (Mikami et al., 1989), where they serve important physiopathological functions; for example, gain-of-function mutations in the *CACNA1C* gene cause Timothy Syndrome (Splawski et al., 2004; Moon et al., 2018). In neurons, two different L-type calcium channels (LTCCs) are expressed: Cav1.2 and Cav1.3 (Hell et al., 1993; Ertel et al., 2000). Cav1.2 is the major calcium channel isoform in neurons, constituting about 80% of neuronal LTCCs (Hell et al., 1993). Cav1.2 participates in learning and memory, drug addiction, and neuronal development (Striessnig et al., 2014). Large-scale genome-wide association studies have shown a strong association between susceptibility to psychiatric disorders and single nucleotide polymorphisms (SNPs) in the *CACNA1C* gene (Bhat et al., 2012). Yet, understanding of Cav1.2 function in the brain and its role in neurodegenerative disease remains limited.

The genetic regulation and channel modulation of Cav1.2 have been studied intensively. At the post-transcriptional level, alternative splicing of Cav1.2 increases protein diversity. Different splice variants have distinct channel properties, with tissue- and disease-specific variability (Wang et al., 2006). At the post-translational level, Cav1.2 is altered by a variety of modifications, which will be further discussed below.

Cav1.2 is an important drug target in the cardiovascular system. DHPs form a class of LTCC blockers and are the most widely prescribed drugs for hypertension and myocardial ischemia (Zamponi et al., 2015). In this review, we summarize the post-translational modifications of Cav1.2 and its role in neurodegenerative diseases, and further discuss the potential of Cav1.2 as a drug target for Alzheimer’s disease (AD) and Parkinson’s disease (PD).

## Structure and Function of Cav1.2 in the CNS

**FIGURE 1:**
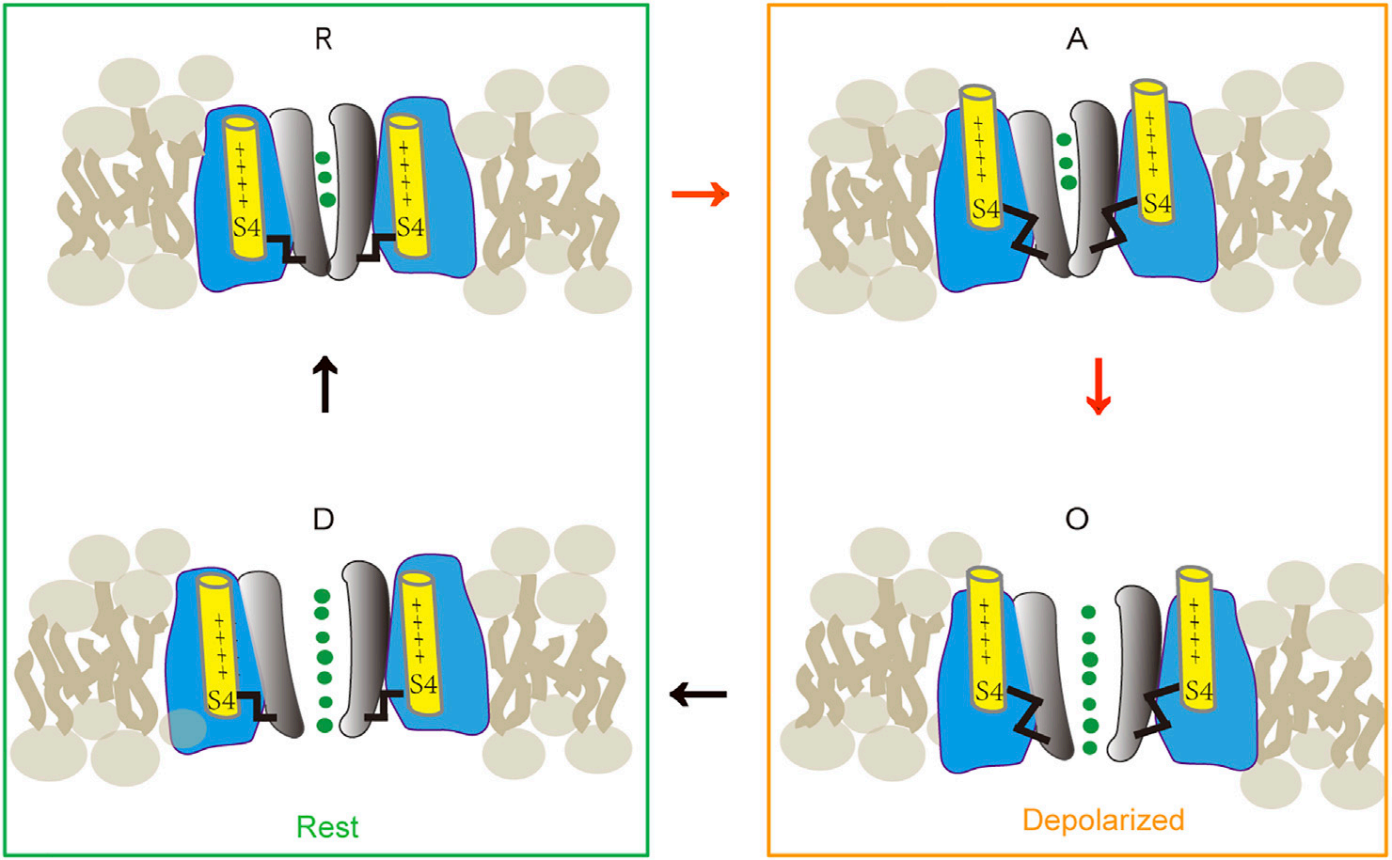
Cav1.2 state transitions during activation [modified after (Beyl et al., 2009) (Hering et al., 2018)]. The channel gating is determined by two functionally distinct processes: a voltage-sensing mechanism and a conducting pore. These two processes defined 4 states: R, at rest, pore is closed and S4 segments in the “down” position lock the pore. A, when depolarized, voltage-sensing mechanism is activated and S4 segments move to the “up” position and release the pore; but the pore remains closed. O, during continuous depolarization, all four S4 segments are in the “up” position; the pore is open. D, when returned to the resting potential, the deactivated voltage-sensing segment moved toward a “down” position while the pore is still open. Subsequently, the pore will transit to its closed conformation and at a resting state.

Voltage-gated calcium channels play an important role in neuronal function (Goonasekera et al., 2012). Cav1.2 is a multi-protein complex. It generally consists of three subunits: a pore-forming subunit α1, a *β* subunit, and an α2δ subunit; in skeletal muscle, a *γ* subunit is also found (Goonasekera et al., 2012). The α1 subunit contains about 2000 amino acid residues, which forms four homologous domains (DI–DIV) connected by intracellular loops (Dai et al., 2009; Alves et al., 2019). Each domain consists of six transmembrane segments: S1 to S6 (Dai et al., 2009; Alves et al., 2019). Of these, S5 and S6 form the pore; and the S4 segment serves as a voltage sensor. The gating mechanism is shown in Figure 1. At rest, the S4 segments stay inward (“down”) under the influence of the electrical field and lock the channel in its closed state. In this state, the S6 helices converge on the intracellular side, preventing ion penetration. When the membrane is depolarized, the S4 segments are released and move outward. The pore will be unlocked when all four S4 segments leave the “down” position. During continuous depolarization, the S6 gate disengages. When all the four S6 segments disengage and are in the “up” position, the pore opens. When returned to the resting potential, the deactivated voltage-sensing segment moved toward a “down” position while the pore is still open. Subsequently, the channel returns to its closed conformation at a rest state (Beyl et al., 2009; Hering et al., 2018).

The α1 subunit is the binding site of most regulators and drugs that act on the channel (Zamponi et al., 2015), whereas the main functions of the other subunits are transportation, anchoring, and regulation (Hofmann et al., 2014). Cav1.2 channels usually require intense depolarization to activate and have long-lasting activity (Hofmann et al., 2014). Ca^2+^ entering through Cav1.2 participates in a series of physiological processes as an important second messenger.

Cav1.2 is distributed universally in the brain. In humans, moderate-to-high mRNA level is detected in the cerebral cortex, the pituitary gland, the amygdala, the basal ganglia, and the cerebellum (Splawski et al., 2004). In mice, the olfactory region, the basal ganglia, the hippocampal formation, the amygdala, and the thalamus show moderate-to-high mRNA level of Cav1.2 (Hell et al., 1993; Splawski et al., 2004; Hetzenauer et al., 2006). At the protein level, the hippocampal formation, the thalamus, and the hypothalamus have moderate-to-strong signal intensity. At the subcellular level in neurons, Cav1.2 is in the soma and at the synapses (Alves et al., 2019).

Cav1.2 plays an important role in the regulation of synaptic plasticity. Researchers found that mice with an inactivated form of the *CACNA1C* gene in the hippocampus and neocortex display severely impaired hippocampus-dependent spatial memory (Moosmang et al., 2005). Cav1.2 is involved in the formation of long-lasting long-term potentiation (LTP) in the hippocampus (Moosmang et al., 2005; Moon et al., 2018; Nanou and Catterall, 2018). Long-lasting LTP needs activation of gene expression and protein synthesis (Malenka and Bear, 2004). The calcium entry from Cav1.2 activates Calmodulin-dependent protein kinase II (CamKII), which binds the C-terminus of Cav1.2; and downstream CamKIV, which phosphorylate CREB and activate downstream gene expression (Cohen et al., 2015). In another pathway, the calcium-regulated phosphatase calcineurin that binds to the C-terminal domain of Cav1.2 is also activated and dephosphorylates the transcription factor NFAT (nuclear factor of activated T-cells), allowing it to translocate into the nucleus and activate gene expression (Murphy et al., 2014). The above signaling cascade increases the synthesis of mRNA encoding synaptic proteins, causing long-lasting changes in synaptic function (Nanou and Catterall, 2018). Moreover, recent studies have found a *β*2-adrenergic receptor and Cav1.2 signaling complex that regulates synaptic plasticity. β2-adrenergic receptors affect calcium channel activity and long-term postsynaptic plasticity through their interactions with the C-terminus of Cav1.2 channels (Qian et al., 2017).

During aging, the viability of Cav1.2 channels increases, leading to high intracellular calcium (Navakkode et al., 2018) that may modulate the processing of amyloid precursor protein (APP) and promote AD pathogenesis (Anekonda and Quinn, 2011). The calcium hypothesis of AD holds that disturbing the intracellular Ca^2+^ balance affects intracellular signal transmission, leading to the formation of Aβ plaques and neurofibrillary tangles, which alter the plasticity of synapses and ultimately lead to the death of neurons (Khachaturian, 1989). Furthermore, Ca^2+^ imbalance promotes the phosphorylation of tau and leads to disordered autophagy in neurons (Anekonda and Quinn, 2011). Endoplasmic reticulum stress (ER stress) and subsequent tau hyperphosphorylation are increased in human chronic traumatic encephalopathy. Administration of docosahexaenoic acid, an endoplasmic reticulum stress inhibitor, lowers intracellular calcium concentration, which results in the decrease of tau hyperphosphorylation and improves cognitive performance (Begum et al., 2014; Lucke-Wold et al., 2016). Separately, salubrinal, a modulator of cellular stress, can reduce neuroinflammation in mice via decreasing ER stress and oxidative stress (Logsdon et al., 2016).

## Post-Translational Modulation of Cav1.2 and its Role in Neurodegenerative Diseases

Post-translational modulation (PTM) is a process that converts synthesized proteins to mature proteins through covalent or enzymatic modifications. These modifications range from the enzymatic hydrolysis of peptide bonds to the covalent addition of specific chemical groups, lipids, carbohydrates, and even entire proteins and amino acid side chains. These chemical modifications after polypeptide chain biosynthesis expand the scope of the amino acid structure and properties, thereby diversifying the structure and function of proteins. PTM can occur at any point and regulates protein activity, localization, and interactions with other molecules (Knorre et al., 2009; Walker and Nestler, 2018).

**FIGURE 2:**
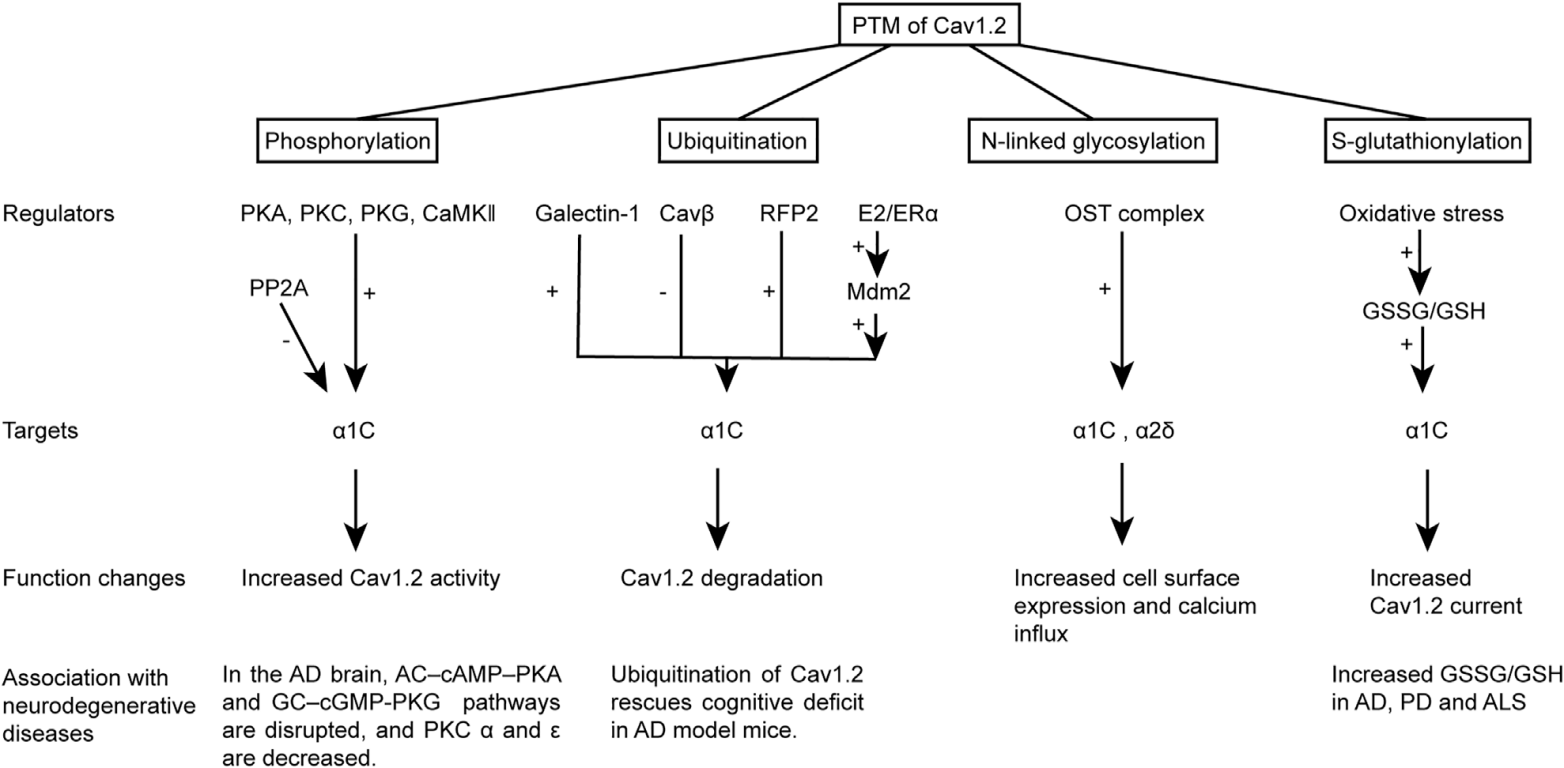
Schematic representation of the PTM of Cav1.2 and its correlation with neurodegenerative diseases. The PTM changes the channel activity, degradation, and cell surface expression of Cav1.2. The PTM of Cav1.2 or the disruption of their regulating pathways was also observed in neurodegenerative diseases.

Cav1.2 undergoes a series of PTMs before it becomes a mature and functional Ca^2+^ channel on the cell surface. These modifications influence the channel properties, trafficking, and location and hence significantly alter the channel function. Cav1.2 modification is dramatically changed in neurodegenerative disease and may be an important component of the pathology (summarized in Figure 2).

### Phosphorylation

Phosphorylation of a molecule is the attachment of a phosphoryl group. Protein phosphorylation is the most abundant post-translational modification in eukaryotes. Phosphorylation can occur on serine, threonine, and tyrosine side chains (often called “residues”) through phosphoester bond formation. Neural cells contain a plethora of protein kinases, protein phosphatases, and phosphorylated proteins, and many of these are essential for the regulation of neuronal morphology and for cell functions as diverse as membrane excitability, secretory processes, cytoskeletal organization, and cellular metabolism.

Phosphorylation of Cav1.2 channels can enhance Ca^2+^ influx four- to six-fold (Sculptoreanu et al., 1993; Kavalali et al., 1997). Cav1.2 channels can be phosphorylated by many protein kinases (PKA, PKC, PKG, and CAMKII) but, in most cases, the sites regulated by these kinases remain uncertain. The identified phosphorylation sites in Cav1.2 are summarized in Table 1 (Perez-Reyes et al., 1992; De Jongh et al., 1996; Gerhardstein et al., 1999; Yang et al., 2005; Grueter et al., 2006; Gui et al., 2006; Yang et al., 2007; Blaich et al., 2010; Fuller et al., 2010; Huttlin et al., 2010; Bachnoff et al., 2011; Brandmayr et al., 2012; Pankonien et al., 2012; Lei et al., 2018; Li et al., 2020; Whitcomb et al., 2020). The central subunit of Cav1.2, α1C, is the major subunit involved in the PKA-mediated increase in channel activity. The α1C subunit is phosphorylated by PKA in intact hippocampal neurons, and a two-fold increase in Ca^2+^ influx has been observed in hippocampal neurons in old rats compared with adult rats, suggestive of increased PKA phosphorylation of Cav1.2 with aging. S1700 phosphorylation plays a greater modulatory role than S1928 phosphorylation in the heart, which is crucial for calcium homeostasis in cardiomyocytes and prevention of heart failure (Yang et al., 2016).

**TABLE1:** Identified phosphorylation sites in Cav1.2 α1C and Cavβ2.

Species	Subunit	Kinases and phosphorylation sites			
		PKA	PKC	CaMKII	PKG
Human	α1	S1898 (Bachnoff et al., 2011)	—	—	—
Mouse	α1	S1897 (Whitcomb et al., 2020)	—	S1512 S1570 (Blaich et al., 2010)	—
		S107, 499, 838, 845, 1680, 1700, 1721, 1744, 1927, 2155 T501, 506 (Huttlin et al., 2010)			
	β2	S200, 202, 203, 211, 214, 510, 545, 522 T215, 549 (Huttlin et al., 2010)			
Rat	α1	—	—	T1604 (Li et al., 2020)	—
	β2	S478 S479 (Perez-Reyes et al., 1992; Bunemann et al., 1999)	—	T498 (Grueter et al., 2006)	—
Rabbit	α1	S1928 (De Jongh et al., 1996) S1700 T1704 (Fuller et al., 2010)	S1928 (Yang et al., 2005)	—	S1928 (Yang et al., 2007)
	β2	S296 (Pankonien et al., 2012) S459 S478 S479 (Gerhardstein et al., 1999)	—	—	S496 (Yang et al., 2007)
Guinea pig	α1	S1574 S1626 S1699 (Lei et al., 2018)	—	T1603 (Li et al., 2020)	—

The publications reporting phosphorylation of a specific amino acid are indicated by a reference in brackets. Notably, amino acids in all the references are not the canonical protein sequences and differ from each other. Please refer to the original publication for more detail.

However, only S1928 has been shown to increase with normal aging in the hippocampus (Davare and Hell, 2003) and S1928 is important for the upregulation of channel activity by PKA. Protein phosphatase 2A (PP2A) constitutively bound to Cav1.2 is required for dephosphorylation of S1928 and subsequent down-regulation of Cav1.2 channel activity (Xu et al., 2010). Similar to PKA, PKC can also phosphorylate α1C at the same site (Weiss et al., 2012). The channel activity of Cav1.2 increases because of the convergence of the two kinases. PKC α and ε expression is decreased with aging in the prefrontal cortex and hippocampus (Perovic et al., 2013), and they are downregulated by Aβ in AD brains (Govoni et al., 1993; Lucke-Wold et al., 2015). PKC signal cascades along with altered calcium homeostasis contribute to the development of NFTs (neurofibrillary tangles) (Lucke-Wold et al., 2014).

The S1928 site is close to the C-terminus of α1C, present only in full-length α1C. With normal aging, there is a clear increase in S1928 phosphorylation in the hippocampus but the general levels of cyclic adenosine monophosphate (cAMP), PP2A, and protein phosphatase 1 (PP1) inhibitors remain unchanged (Davare and Hell, 2003). The dentate gyrus is the major region in the hippocampus where S1928 phosphorylation occurs; no significant changes are observed in other areas of the hippocampus (Núñez-Santana et al., 2014). S1928 phosphorylation by A-kinase-anchoring protein (AKAP)-anchored PKA plays an essential role in enhancing Cav1.2 channel activity and vasoconstriction under conditions of high glucose or in diabetes (Nystoriak et al., 2017). The level of cAMP is upregulated in cerebral vessels in AD hippocampus and is associated with vascular *β*-amyloid peptide (Aβ) (Martínez et al., 2001). It is well established that patients with type 2 diabetes have a higher incidence of cognitive decline and morbidity of AD than the general population (Surguchov, 2020), suggestive of a link with changes in Cav1.2 activity. In the AD brain, preclinical and neuropathological data suggest that both adenyl cyclase (AC)–cAMP–PKA and guanylate cyclase (GC)–cGMP–PKG signaling are disrupted. Overall PKA activity and nuclear PKA activity appear to be suppressed in AD (Sanders and Rajagopal, 2020), which may lead to abnormal changes in Cav1.2 phosphorylation state. Furthermore, the mechanism by which the β2-adrenergic receptor (β2AR) stimulates Cav1.2 channel activity depends on S1928 phosphorylation and constitutes a critical component of the molecular mechanism underlying stable and prolonged theta-tetanus-induced LTP (Qian et al., 2017). Multiple phosphorylation sites have been found in the C-terminal domain of the Cav1.2 β subunit *in vitro*. However, C-terminal knock-out mice survive with no apparent physiological deficits and, most importantly, show normal function of Cav1.2 in ventricular myocytes. Thus, the phosphorylation sites on the Cav1.2 β subunit may not have essential functional roles *in vivo*.

### Ubiquitination

Ubiquitin (UB) is a highly conserved small protein that is found in all eukaryotic cells, from single-celled yeast to humans. Its main function is to mark proteins to be degraded by 26S proteasome (Lam et al., 2000; Bennett et al., 2005; Swatek and Komander, 2016). UB binds covalently to the lysine residue of the substrate protein and the ubiquitin-labeled protein is identified and rapidly degraded. Briefly, this process requires the sequential action of three enzymes (Swatek and Komander, 2016). The C-terminal glycine residue of ubiquitin is activated by E1. Next, activated ubiquitin is transferred to an active cysteine residue of E2. Finally, ubiquitin links its C-terminus to an ε-amino group of the substrate protein’s lysine residues (Hershko and Ciechanover, 1998). In a nutshell, ubiquitination is a dynamic, multifaceted post-translational modification that is involved in nearly all physiological processes (Swatek and Komander, 2016). An abnormal UB signal is closely related to neurodegeneration.

Neurodegenerative diseases are characterized by the loss of neurons in the brain or spinal cord. Most samples from patients with neurodegenerative diseases are immunoreactive for anti-UB antibodies (Popovic et al., 2014) and abnormalities of the UB-dependent degradation systems and aggregation formation are associated with neurodegeneration (Hershko and Ciechanover, 1998; Lam et al., 2000; Bennett et al., 2005; Hara et al., 2006). In PD, α-synuclein in Lewy bodies (a diagnostic marker of PD) is modified by ubiquitin at lysines 77 and 78 (Popovic et al., 2014). Ubiquitination likely increases the aggregation and neurotoxicity of α-synuclein in cultured human dopaminergic cells (Popovic et al., 2014). In AD, a typical aggregate is ubiquitinated tau protein (Popovic et al., 2014). Thus, UB-dependent degradation systems, such as the UB-proteasome system and autophagy, likely play a role in the pathogenesis of these neurodegenerative diseases (Bennett et al., 2005; Popovic et al., 2014).

The ubiquitin–proteasome system (UPS) is closely linked to Cav1.2 degradation (Felix and Weiss, 2017). UB protein has seven lysine residues at positions 6, 11, 27, 29, 33, 48, and 63 (Ikeda and Dikic, 2008; Chen and Sun, 2009). Among these, K6/K29 take part in Cav1.2 degradation (Lai et al., 2019). E3 specifically recognizes the target proteins’ lysine residue and tags it for degradation by the proteasome (Hershko and Ciechanover, 1998). Recent studies have shown that the Cavβ subunit may serve as a molecular switch that prevents the Cav1.2 α subunit from ubiquitination by the RFP2 ubiquitin ligase and subsequent transfer of Cav1.2 channels to the endoplasmic reticulum associated protein degradation (ERAD) complex; thus, the Cavβ subunit protects Cav1.2 channels from proteasomal degradation (Felix and Weiss, 2017). Separately, Galectin-1 acts as a negative Cav1.2 channel regulator by binding to the Cav1.2 I–II loop and exposing the lysine residues inside the loop to polyubiquitination and ERAD degradation, ultimately inhibiting channel function (Hu et al., 2018; Loh et al., 2020). Furthermore, in ovariectomized APP/PS1 mice (an AD animal model), systemic administration of E2 (17β-estradiol) or the estrogen receptor α (ERα) agonist propylpyrazoletriol (PPT) increased ubiquitination of Cav1.2 in the brain, reversed elevated levels of Cav1.2 protein, and improved cognitive functioning. The binding of the E3 ligase Mdm2 with Cav1.2 is promoted by activating ERα. In Mdm2-overexpressing neurons, the intensity of Cav1.2 decreased significantly. These results suggest that Mdm2-related ubiquitination is critical for ERα regulation of Cav1.2 protein levels and that a reduction in Cav1.2 protein levels may contribute to ERα-induced cognitive improvements (Lai et al., 2019).

### N-Linked Glycosylation

N-linked glycosylation is a co-translational or post-translational modification of new peptide chains in which oligosaccharides are connected to the amide of asparagine residues. N-linked glycosylation can be divided into high mannose, compound, and heterozygous types. N-linked glycosylation consists of three main steps: synthesis, transfer, and modification. Synthesis and transfer of N-linked glycosylation are carried out in the endoplasmic reticulum, whereas modification occurs in both the endoplasmic reticulum and the Golgi matrix. This progress is necessary for membrane trafficking and protein expression on the cell surface. Recent studies showed that external glucose level alters N-glycosylation (Liu et al., 2014; Villacrés et al., 2015). There are four potential N-glycosylation sites in the rabbit Cav1.2: N124, N299, N1359, and N1410. The double mutant (N124, 299Q) showed a positive shift in the voltage-dependent gating curve; and the quadruple mutant (QM; N124, 299, 1,359, 1410Q) showed a positive shift in the voltage-dependent gating curve as well as a reduction of peak current. The weaker surface fluorescence intensity of QM suggested its lower surface expression than wild-type Cav1.2 (Park et al., 2015).

The α2δ subunit, an integral component of Cav1.2, is highly N-glycosylated by a 30-kDa oligosaccharide (Marais et al., 2001). Mutation of only 6/16 asparagine glycosylation sites was sufficient to decrease cell surface expression and protein stability of α2δ1 subunit, as well as α2δ1-mediated peak current density and voltage-dependent gating of the α1C subunit. Single mutation N663Q and double mutations N348Q/N468Q, N348Q/N812Q, and N468Q/N812Q decreased protein stability and abolished cell surface expression of α2δ1 as well as the α2δ1-induced up-regulation of Cav1.2 currents (Tétreault et al., 2016). However, it is still not clear whether N-glycosylation of Cav1.2 contributes to the mechanism of Ca^2+^ interruption in neurodegenerative diseases.

### S-Glutathionylation

S-glutathionylation is a process in which glutathione forms a disulfide bond with cysteine residues of the target protein, and is a major redox-mediated thiol modulation. Oxidative stress facilitates S-glutathionylation. The ratio of reduced and oxidized glutathione (GSH/GSSG) is important for S-glutathionylation. Glutathionylation is a reversible redox modification: it directly changes the redox state of Cav1.2 and increases calcium influx (Tang et al., 2011). However, this process is considered an oxidant-mediated reaction with low specificity for target proteins. C543 in the cytoplasmic I-II loop is the major glutathiolation target in hCav1.2. C543S mutation alters post-translational folding and shifts the channel open probability, which may lead to the onset of disease pathology (Muralidharan et al., 2016). Inflammation and ROS are known to be critical pathological manifestations of neurodegenerative diseases. Moreover, imbalance of glutathione homeostasis and dysregulation in glutathione-dependent enzyme activities are implicated in the induction and progression of neurodegenerative diseases, including AD, PD, and ALS. Therefore, impaired S-glutathionylation of Cav1.2 may contribute to the pathology of neurodegenerative diseases.

## Cav1.2 as a Potential Drug Target in Neurodegenerative Diseases

Cav1.2 is a classical drug target for cardiovascular disease. Members of the dihydropyridine family of calcium channel blockers (DHPs) have been used as first-line drugs for hypertension and myocardial ischemia for decades, including amlodipine, felodipine, and nifedipine (Zamponi et al., 2015). The sensitivity of LTCCs to DHPs varies in different tissues. Cav1.2 is more sensitive to DHPs than Cav1.3 and Cav1.4 (Xu and Lipscombe, 2001). The splice variants of Cav1.2 in arterial smooth muscle are more sensitive to DHPs than those in the myocardium (Liao et al., 2004; Cheng et al., 2009).

Because of the pathophysiological role of Cav1.2 in neurodegenerative disease, DHPs have been repurposed as a treatment for these diseases. DHPs have at least two advantages as drugs for CNS indications: safety and penetration of the blood–brain barrier (BBB). At therapeutic doses, no obvious side effects were observed for muscle function, hearing, CNS function, or insulin secretion, where LTCCs exert important functions (Levine et al., 2007). Several DHPs can cross the BBB in some species, including humans (Allen et al., 1983; Uchida et al., 1997). Intracerebral drug delivery methods have also improved recently (Patel et al., 2009; Lu et al., 2014).

Because of the known role of Cav1.2 in cognition and the imbalance in Ca^2+^ homeostasis found in AD, DHPs have been repurposed for AD treatment. In a survey of investigating the association between DHP or non-DHP calcium channel blocker and risk of developing AD or mortality, researchers found that the use of DHP did not reduce risk of AD but showed lower relative risk (Yasar et al., 2005). *In vitro*, nilvadipine, nitrendipine, and amlodipine reduced Aβ accumulation by affecting the production and clearance of Aβ. *In vivo*, nilvadipine and nitrendipine reduced Aβ deposition. In transgenic mouse models of AD (Tg APPsw (Tg2576) and Tg PS1/APPsw), chronic nilvadipine treatment resulted in lower Aβ levels and improved learning and spatial memory (Paris et al., 2011). These results suggest that some DHPs have significant benefits in the treatment of AD. Nilvadipine can also delay the degeneration of cognitive function in AD patients (Hanyu et al., 2007; Matsuda et al., 2008). Nitrendipine treatment reduced the risk of dementia by 55% in hypertensive patients compared with a control group (Forette et al., 2002). Since improvements in cognition are observed with non-DHP drugs like ACEI and thiazide (Bellew et al., 2004; Hanon and Forette, 2004; Fournier et al., 2009; Duron and Hanon, 2010), the protective effects of nivadipine and nitrendipine do not seem to be related to their antihypertensive effects. Although nilvadipine and nitrendipine have protective effects, their effectiveness depends on the severity of AD (Paris et al., 2011). After nilvadipine treatment, the very mild AD group showed less cognition decline whereas the moderate AD group showed greater cognition decline compared with their respective placebo-treated controls (Abdullah et al., 2020). This study suggests that AD severity affects the treatment results and nilvadipine may be restricted to patients with mild AD in the future.

The pathological mechanisms underlying PD are not yet clear. Symptomatic treatments are aimed at relieving deficits in motor symptoms and improving quality of life (Schulz et al., 2016; Obeso et al., 2017). Currently, pharmacotherapy includes dopamine mimetics (levodopa), synergists of levodopa (selegiline, carbidopa), dopamine receptor agonists (bromocriptine), dopamine-releasing drugs (amantadine), and anticholinergic drugs (trihexsyphenidyl). Neurosurgery and supportive treatments have been used clinically for many years (Oertel and Schulz, 2016; Schulz et al., 2016). However, none of these treatment methods can prevent or slow the progression of PD and the side effects of the treatments often limit the long-term benefits of symptomatic therapies. However, there are a few different drugs currently in preclinical trials. Because of LTCC-mediated Ca^2+^ load in SNc dopaminergic neurons, DHPs are considered for PD treatment. Studies have shown that isradipine has a significant neuroprotective effect on substantia nigral dopaminergic neurons in an MPTP-induced animal model of PD (Kupsch et al., 1995; Singh et al., 2016; Wang et al., 2017) and partially restores dopamine content in the striatum (Wang et al., 2017). Another DHP, nifedipine, was reported to improve apomorphine-induced rotation behavior in 6-OHDA-lesioned rats (Wang et al., 2012).

In humans, the ongoing phase III clinical study STEADY-PD is investigating the potential of the LTCC blocker isradipine for treatment of PD. Although the study showed that long-term treatment with immediate-release isradipine did not slow the clinical progression of early-stage PD, it did modestly decrease cumulative levodopa equivalent dose and the time needed for antiparkinsonian treatment (McFarthing and Simuni, 2019; Parkinson Study Group STEADY-PD III Investigators, 2020; Venuto et al., 2021). According to epidemiological studies (Becker et al., 2008; Ritz et al., 2010; Pasternak et al., 2012; Lee et al., 2014) and meta-analyses (Gudala et al., 2015; Lang et al., 2015; Mullapudi et al., 2016), patients treated with DHPs have a reduced risk of PD. Although DHPs have a history of safe use, the drug release time should be prolonged to avoid activation of the sympathetic nervous system, accompanied by reflex tachycardia and high cardiac oxygen consumption, flushing, hypotension, and headache (Carrara et al., 1994; Johnson et al., 2005). In some countries, extended-release formulations of isradipine are available and are already in phase II clinical trials in PD patients (Parkinson Study Group, 2013).

Other potential treatment strategies remain to be studied. Previous data show that the basal level of Cav1.2 in the hippocampus and cortex of ovariectomized APP/PS1 mice is significantly higher than that of wild-type mice. E2 or PPT could reverse this increased basal level of Cav1.2 by promoting the ubiquitination and degradation of Cav1.2 (Lai et al., 2019). Thus, ERα agonists (propylpyrazoletriol, dienestrol) may effectively alleviate the symptoms of AD. Hu et al. used a Tat-e9c peptide to compete for the Galectin-1 binding site on Cav1.2 and interfere with its ubiquitination and degradation (Hu et al., 2018), but whether Cavβ-derived peptides can be used to promote Cav1.2 degradation in the brain needs further study. The biggest concerns would be how to transport the peptide across the BBB and how to reduce the side effects in the cardiovascular system.

## Conclusion

Cav1.2 plays important roles in the cardiovascular system, the CNS, and endocrine glands. In the brain, it mediates learning and memory, drug addiction, and neuronal development. Cav1.2 undergoes a variety of post-translational modifications, which are altered in neurodegenerative disease states. Recently identified modifications, such as S-nitrosylation, and their role in pathology require further study.

DHPs are widely prescribed for hypertension and myocardial ischemia and have been repurposed for use in neurodegenerative diseases including AD and PD. Several clinical trials show promising outcomes (summarized in Table 2). Although clinical studies have shown that DHPs have protective effects on neurodegenerative diseases, there are several issues with using DHPs to treat neurodegenerative diseases. First, achieving the requisite drug concentrations in the brain while avoiding fluctuations in blood pressure and cardiac function is a challenge. This may be addressed by the development of new drug-delivery strategies. Second, the relative lack of selectivity of DHPs is a big concern for their use in the CNS; unwanted effects may arise from antagonism of Cav1.3 channels. Furthermore, the universal expression of Cav1.2 may result in DHP side effects on normal brain functions. Further studies on channel modulation and more-specific methods of altering Cav1.2 channel function may lead to better therapeutic strategies for neurodegenerative diseases.

**TABLE 2:** Summary of clinical trials and surveys on the effects of DHPs in neurodegenerative diseases

Drug	Stage	Duration	Dose	Number	Indication	Results	References
Nitrendipine	Survey	3.9 years	10–40 mg/d	148	AD	Treatment with nitrendipine reduced the risk of dementia by 55%	Forette et al. (2002)
DHP	Survey	2 years	—	1,092	AD	Relative risks were low with the DHP group	Yasar et al. (2005)
DHP	Survey	2 years	—	173	PD	Exposure to DHP reduced the risk of incidence, particularly in older patients, and mortality	Pasternak et al. (2012)
Isradipine	Clinical phase III	36 m	10 mg/d	336	PD	Treatment with isradipine did not slow the clinical progression of early-stage PD	Parkinson Study Group STEADY-PD III Investigators (2020)
Isradipine	Clinical phase III	36 m	10 mg/d	166	PD	Exposure to DHP reduced the risk of needing antiparkinsonian treatment	Venuto et al. (2021)
Isradipine	Clinical phase III	36 m	10 mg/d	162	PD	Treatment with isradipine slows progression of PD disability	https://clinicaltrials.gov/ct2/show/study/NCT02168842?term=isradipine&cond=Parkinson%27s+disease&draw=2&rank=2

## References

[B1] Abdullah L. Crawford F. Tsolaki M. Börjesson-Hanson A. Olde Rikkert M. Pasquier F. (2020). The Influence of Baseline Alzheimer's Disease Severity on Cognitive Decline and CSF Biomarkers in the NILVAD Trial. Front. Neurol. 11, 149. 10.3389/fneur.2020.00149 32210906 PMC7067750

[B2] Allen G. S. Ahn H. S. Preziosi T. J. Battye R. Boone S. C. Boone S. C. (1983). Cerebral Arterial Spasm-Aa Controlled Trial of Nimodipine in Patients with Subarachnoid Hemorrhage. N. Engl. J. Med. 308 (11), 619–624. 10.1056/NEJM198303173081103 6338383

[B3] Alves V. S. Alves-Silva H. S. Orts D. J. B. Ribeiro-Silva L. Arcisio-Miranda M. Oliveira F. A. (2019). Calcium Signaling in Neurons and Glial Cells: Role of Cav1 Channels. Neuroscience 421, 95–111. 10.1016/j.neuroscience.2019.09.041 31678346

[B4] Anekonda T. S. Quinn J. F. (2011). Calcium Channel Blocking as a Therapeutic Strategy for Alzheimer's Disease: the Case for Isradipine. Biochim. Biophys. Acta 1812 (12), 1584–1590. 10.1016/j.bbadis.2011.08.013 21925266 PMC3275089

[B5] Bachnoff N. Cohen-Kutner M. Atlas D. (2011). The Involvement of Ser1898 of the Human L-type Calcium Channel in Evoked Secretion. Int. J. Endocrinol. 2011, 746482. 10.1155/2011/746482 22216029 PMC3246732

[B6] Becker C. Jick S. S. Meier C. R. (2008). Use of Antihypertensives and the Risk of Parkinson Disease. Neurology 70 (16 Pt 2), 1438–1444. 10.1212/01.wnl.0000303818.38960.44 18256367

[B7] Begum G. Yan H. Q. Li L. Singh A. Dixon C. E. Sun D. (2014). Docosahexaenoic Acid Reduces ER Stress and Abnormal Protein Accumulation and Improves Neuronal Function Following Traumatic Brain Injury. J. Neurosci. 34 (10), 3743–3755. 10.1523/JNEUROSCI.2872-13.2014 24599472 PMC6608987

[B8] Bellew K. M. Pigeon J. G. Stang P. E. Fleischman W. Gardner R. M. Baker W. W. (2004). Hypertension and the Rate of Cognitive Decline in Patients with Dementia of the Alzheimer Type. Alzheimer Dis. Assoc. Disord. 18 (4), 208–213. 15592132

[B9] Bennett E. J. Bence N. F. Jayakumar R. Kopito R. R. (2005). Global Impairment of the Ubiquitin-Proteasome System by Nuclear or Cytoplasmic Protein Aggregates Precedes Inclusion Body Formation. Mol. Cell 17 (3), 351–365. 10.1016/j.molcel.2004.12.021 15694337

[B10] Beyl S. Kügler P. Kudrnac M. Hohaus A. Hering S. Timin E. (2009). Different Pathways for Activation and Deactivation in CaV1.2: a Minimal Gating Model. J. Gen. Physiol. 134 (3), 231–241. 10.1085/jgp.200910272 19687230 PMC2737230

[B11] Bhat S. Dao D. T. Terrillion C. E. Arad M. Smith R. J. Soldatov N. M. (2012). CACNA1C (Cav1.2) in the Pathophysiology of Psychiatric Disease. Prog. Neurobiol. 99 (1), 1–14. 10.1016/j.pneurobio.2012.06.001 22705413 PMC3459072

[B12] Blaich A. Welling A. Fischer S. Wegener J. W. Köstner K. Hofmann F. (2010). Facilitation of Murine Cardiac L-type Ca(v)1.2 Channel Is Modulated by Calmodulin Kinase II-dependent Phosphorylation of S1512 and S1570. Proc. Natl. Acad. Sci. U S A. 107 (22), 10285–10289. 10.1073/pnas.0914287107 20479240 PMC2890469

[B13] Brandmayr J. Poomvanicha M. Domes K. Ding J. Blaich A. Wegener J. W. (2012). Deletion of the C-Terminal Phosphorylation Sites in the Cardiac β-subunit Does Not Affect the Basic β-adrenergic Response of the Heart and the Ca(v)1.2 Channel. J. Biol. Chem. 287 (27), 22584–22592. 10.1074/jbc.M112.366484 22589548 PMC3391128

[B120] Bunemann M. Gerhardstein B. L. Gao T. Hosey M. M. (1999). Functional Regulation of L-Type Calcium Channels Via Protein Kinase A-Mediated Phosphorylation of the Beta(2) Subunit. J. Biol. Chem. 274 (48), 33851–33854. 10.1074/jbc.274.48.33851 10567342

[B14] Carrara V. Porchet H. Dayer P. (1994). Influence of Input Rates on (+/-)-isradipine Haemodynamics and Concentration-Effect Relationship in Healthy Volunteers. Eur. J. Clin. Pharmacol. 46 (1), 29–33. 10.1007/BF00195912 8005183

[B15] Chen Z. J. Sun L. J. (2009). Nonproteolytic Functions of Ubiquitin in Cell Signaling. Mol. Cell 33 (3), 275–286. 10.1016/j.molcel.2009.01.014 19217402

[B16] Cheng X. Pachuau J. Blaskova E. Asuncion-Chin M. Liu J. Dopico A. M. (2009). Alternative Splicing of Cav1.2 Channel Exons in Smooth Muscle Cells of Resistance-Size Arteries Generates Currents with Unique Electrophysiological Properties. Am. J. Physiol. Heart Circ. Physiol. 297 (2), H680–H688. 10.1152/ajpheart.00109.2009 19502562 PMC2724194

[B17] Cohen S. M. Li B. Tsien R. W. Ma H. (2015). Evolutionary and Functional Perspectives on Signaling from Neuronal Surface to Nucleus. Biochem. Biophys. Res. Commun. 460 (1), 88–99. 10.1016/j.bbrc.2015.02.146 25998737 PMC4701207

[B18] Dai S. Hall D. D. Hell J. W. (2009). Supramolecular Assemblies and Localized Regulation of Voltage-Gated Ion Channels. Physiol. Rev. 89 (2), 411–452. 10.1152/physrev.00029.2007 19342611 PMC2733249

[B19] Davare M. A. Hell J. W. (2003). Increased Phosphorylation of the Neuronal L-type Ca(2+) Channel Ca(v)1.2 during Aging. Proc. Natl. Acad. Sci. U S A. 100 (26), 16018–16023. 10.1073/pnas.2236970100 14665691 PMC307685

[B20] De Jongh K. S. Murphy B. J. Colvin A. A. Hell J. W. Takahashi M. Catterall W. A. (1996). Specific Phosphorylation of a Site in the Full-Length Form of the Alpha 1 Subunit of the Cardiac L-type Calcium Channel by Adenosine 3',5'-cyclic Monophosphate-dependent Protein Kinase. Biochemistry 35 (32), 10392–10402. 10.1021/bi953023c 8756695

[B21] Duron E. Hanon O. (2010). Antihypertensive Treatments, Cognitive Decline, and Dementia. J. Alzheimers Dis. 20 (3), 903–914. 10.3233/JAD-2010-091552 20182022

[B22] Ertel E. A. Campbell K. P. Harpold M. M. Hofmann F. Mori Y. Perez-Reyes E. (2000). Nomenclature of Voltage-Gated Calcium Channels. Neuron 25 (3), 533–535. 10.1016/s0896-6273(00)81057-0 10774722

[B23] Felix R. Weiss N. (2017). Ubiquitination and Proteasome-Mediated Degradation of Voltage-Gated Ca2+ Channels and Potential Pathophysiological Implications. Gen. Physiol. Biophys. 36 (1), 1–5. 10.4149/gpb_2016037 27787228

[B24] Forette F. Seux M. L. Staessen J. A. Thijs L. Babarskiene M. R. Babeanu S. (2002). The Prevention of Dementia with Antihypertensive Treatment: New Evidence from the Systolic Hypertension in Europe (Syst-Eur) Study. Arch. Intern. Med. 162 (18), 2046–2052. 10.1001/archinte.162.18.2046 12374512

[B25] Fournier A. Oprisiu-Fournier R. Serot J. M. Godefroy O. Achard J. M. Faure S. (2009). Prevention of Dementia by Antihypertensive Drugs: How AT1-Receptor-Blockers and Dihydropyridines Better Prevent Dementia in Hypertensive Patients Than Thiazides and ACE-Inhibitors. Expert Rev. Neurother 9 (9), 1413–1431. 10.1586/ern.09.89 19769454

[B26] Fuller M. D. Emrick M. A. Sadilek M. Scheuer T. Catterall W. A. (2010). Molecular Mechanism of Calcium Channel Regulation in the Fight-Or-Flight Response. Sci. Signal. 3 (141), ra70. 10.1126/scisignal.2001152 20876873 PMC3063709

[B27] Gerhardstein B. L. Puri T. S. Chien A. J. Hosey M. M. (1999). Identification of the Sites Phosphorylated by Cyclic AMP-dependent Protein Kinase on the Beta 2 Subunit of L-type Voltage-dependent Calcium Channels. Biochemistry 38 (32), 10361–10370. 10.1021/bi990896o 10441130

[B28] Goonasekera S. A. Hammer K. Auger-Messier M. Bodi I. Chen X. Zhang H. (2012). Decreased Cardiac L-type Ca²⁺ Channel Activity Induces Hypertrophy and Heart Failure in Mice. J. Clin. Invest. 122 (1), 280–290. 10.1172/JCI58227 22133878 PMC3248289

[B29] Govoni S. Bergamaschi S. Racchi M. Battaini F. Binetti G. Bianchetti A. (1993). Cytosol Protein Kinase C Downregulation in Fibroblasts from Alzheimer's Disease Patients. Neurology 43 (12), 2581–2586. 10.1212/wnl.43.12.2581 8255461

[B30] Grueter C. E. Abiria S. A. Dzhura I. Wu Y. Ham A. J. Mohler P. J. (2006). L-type Ca2+ Channel Facilitation Mediated by Phosphorylation of the Beta Subunit by CaMKII. Mol. Cell 23 (5), 641–650. 10.1016/j.molcel.2006.07.006 16949361

[B31] Gudala K. Kanukula R. Bansal D. (2015). Reduced Risk of Parkinson's Disease in Users of Calcium Channel Blockers: A Meta-Analysis. Int. J. Chronic Dis. 2015, 697404. 10.1155/2015/697404 26464872 PMC4590944

[B32] Gui P. Wu X. Ling S. Stotz S. C. Winkfein R. J. Wilson E. (2006). Integrin Receptor Activation Triggers Converging Regulation of Cav1.2 Calcium Channels by C-Src and Protein Kinase A Pathways. J. Biol. Chem. 281 (20), 14015–14025. 10.1074/jbc.M600433200 16554304

[B33] Hanon O. Forette F. (2004). Prevention of Dementia: Lessons from SYST-EUR and PROGRESS. J. Neurol. Sci. 226 (1-2), 71–74. 10.1016/j.jns.2004.09.015 15537524

[B34] Hanyu H. Hirao K. Shimizu S. Sato T. Kiuchi A. Iwamoto T. (2007). Nilvadipine Prevents Cognitive Decline of Patients with Mild Cognitive Impairment. Int. J. Geriatr. Psychiatry 22 (12), 1264–1266. 10.1002/gps.1851 18033677

[B35] Hara T. Nakamura K. Matsui M. Yamamoto A. Nakahara Y. Suzuki-Migishima R. (2006). Suppression of Basal Autophagy in Neural Cells Causes Neurodegenerative Disease in Mice. Nature 441 (7095), 885–889. 10.1038/nature04724 16625204

[B36] Hell J. W. Westenbroek R. E. Warner C. Ahlijanian M. K. Prystay W. Gilbert M. M. (1993). Identification and Differential Subcellular Localization of the Neuronal Class C and Class D L-type Calcium Channel Alpha 1 Subunits. J. Cell Biol 123 (4), 949–962. 10.1083/jcb.123.4.949 8227151 PMC2200142

[B37] Hering S. Zangerl-Plessl E. M. Beyl S. Hohaus A. Andranovits S. Timin E. N. (2018). Calcium Channel Gating. Pflugers Arch. 470 (9), 1291–1309. 10.1007/s00424-018-2163-7 29951751 PMC6096772

[B38] Hershko A. Ciechanover A. (1998). The Ubiquitin System. Annu. Rev. Biochem. 67, 425–479. 10.1146/annurev.biochem.67.1.425 9759494

[B39] Hetzenauer A. Sinnegger-Brauns M. J. Striessnig J. Singewald N. (2006). Brain Activation Pattern Induced by Stimulation of L-type Ca2+-Channels: Contribution of Ca(V)1.3 and Ca(V)1.2 Isoforms. Neuroscience 139 (3), 1005–1015. 10.1016/j.neuroscience.2006.01.059 16542784

[B40] Hofmann F. Flockerzi V. Kahl S. Wegener J. W. (2014). L-type CaV1.2 Calcium Channels: from *In Vitro* Findings to *In Vivo* Function. Physiol. Rev. 94 (1), 303–326. 10.1152/physrev.00016.2013 24382889

[B41] Hu Z. Li G. Wang J. W. Chong S. Y. Yu D. Wang X. (2018). Regulation of Blood Pressure by Targeting CaV1.2-Galectin-1 Protein Interaction. Circulation 138 (14), 1431–1445. 10.1161/CIRCULATIONAHA.117.031231 29650545 PMC6185826

[B42] Huttlin E. L. Jedrychowski M. P. Elias J. E. Goswami T. Rad R. Beausoleil S. A. (2010). A Tissue-specific Atlas of Mouse Protein Phosphorylation and Expression. Cell 143 (7), 1174–1189. 10.1016/j.cell.2010.12.001 21183079 PMC3035969

[B43] Ikeda F. Dikic I. (2008). Atypical Ubiquitin Chains: New Molecular Signals. 'Protein Modifications: Beyond the Usual Suspects' Review Series. EMBO Rep. 9 (6), 536–542. 10.1038/embor.2008.93 18516089 PMC2427391

[B44] Johnson B. A. Javors M. A. Lam Y. W. Wells L. T. Tiouririne M. Roache J. D. (2005). Kinetic and Cardiovascular Comparison of Immediate-Release Isradipine and Sustained-Release Isradipine Among Non-treatment-seeking, Cocaine-dependent Individuals. Prog. Neuropsychopharmacol. Biol. Psychiatry 29 (1), 15–20. 10.1016/j.pnpbp.2004.08.014 15610940

[B45] Kavalali E. T. Hwang K. S. Plummer M. R. (1997). cAMP-Dependent Enhancement of Dihydropyridine-Sensitive Calcium Channel Availability in Hippocampal Neurons. J. Neurosci. 17 (14), 5334–5348. 10.1523/jneurosci.17-14-05334.1997 9204918 PMC6793811

[B46] Khachaturian Z. S. (1989). Calcium, Membranes, Aging, and Alzheimer's Disease. Introduction and Overview. Ann. N. Y Acad. Sci. 568, 1–4. 10.1111/j.1749-6632.1989.tb12485.x 2629579

[B47] Knorre D. G. Kudryashova N. V. Godovikova T. S. (2009). Chemical and Functional Aspects of Posttranslational Modification of Proteins. Acta Naturae 1 (3), 29–51. 10.32607/actanaturae.10755 22649613 PMC3347534

[B48] Kupsch A. Gerlach M. Pupeter S. C. Sautter J. Dirr A. Arnold G. (1995). Pretreatment with Nimodipine Prevents MPTP-Induced Neurotoxicity at the Nigral, but Not at the Striatal Level in Mice. Neuroreport 6 (4), 621–625. 10.1097/00001756-199503000-00009 7605913

[B49] Lai Y. J. Zhu B. L. Sun F. Luo D. Ma Y. L. Luo B. (2019). Estrogen Receptor α Promotes Cav1.2 Ubiquitination and Degradation in Neuronal Cells and in APP/PS1 Mice. Aging Cell 18 (4), e12961. 10.1111/acel.12961 31012223 PMC6612642

[B50] Lam Y. A. Pickart C. M. Alban A. Landon M. Jamieson C. Ramage R. (2000). Inhibition of the Ubiquitin-Proteasome System in Alzheimer's Disease. Proc. Natl. Acad. Sci. U S A. 97 (18), 9902–9906. 10.1073/pnas.170173897 10944193 PMC27620

[B51] Lang Y. Gong D. Fan Y. (2015). Calcium Channel Blocker Use and Risk of Parkinson's Disease: a Meta-Analysis. Pharmacoepidemiol. Drug Saf. 24 (6), 559–566. 10.1002/pds.3781 25845582

[B52] Lee Y. C. Lin C. H. Wu R. M. Lin J. W. Chang C. H. Lai M. S. (2014). Antihypertensive Agents and Risk of Parkinson's Disease: a Nationwide Cohort Study. PLoS One 9 (6), e98961. 10.1371/journal.pone.0098961 24910980 PMC4049613

[B53] Lei M. Xu J. Gao Q. Minobe E. Kameyama M. Hao L. (2018). PKA Phosphorylation of Cav1.2 Channel Modulates the Interaction of Calmodulin with the C Terminal Tail of the Channel. J. Pharmacol. Sci. 137 (2), 187–194. 10.1016/j.jphs.2018.05.010 30042022

[B54] Levine M. Boyer E. W. Pozner C. N. Geib A. J. Thomsen T. Mick N. (2007). Assessment of Hyperglycemia after Calcium Channel Blocker Overdoses Involving Diltiazem or Verapamil. Crit. Care Med. 35 (9), 2071–2075. 10.1097/01.ccm.0000278916.04569.23 17855820

[B55] Li J. Wang S. Zhang J. Liu Y. Zheng X. Ding F. (2020). The CaMKII Phosphorylation Site Thr1604 in the CaV1.2 Channel Is Involved in Pathological Myocardial Hypertrophy in Rats. Channels (Austin) 14 (1), 151–162. 10.1080/19336950.2020.1750189 32290730 PMC7188351

[B56] Liao P. Yu D. Lu S. Tang Z. Liang M. C. Zeng S. (2004). Smooth Muscle-Selective Alternatively Spliced Exon Generates Functional Variation in Cav1.2 Calcium Channels. J. Biol. Chem. 279 (48), 50329–50335. 10.1074/jbc.M409436200 15381693

[B57] Liu B. Spearman M. Doering J. Lattová E. Perreault H. Butler M. (2014). The Availability of Glucose to CHO Cells Affects the Intracellular Lipid-Linked Oligosaccharide Distribution, Site Occupancy and the N-Glycosylation Profile of a Monoclonal Antibody. J. Biotechnol. 170, 17–27. 10.1016/j.jbiotec.2013.11.007 24286971

[B58] Logsdon A. F. Lucke-Wold B. P. Nguyen L. Matsumoto R. R. Turner R. C. Rosen C. L. (2016). Salubrinal Reduces Oxidative Stress, Neuroinflammation and Impulsive-like Behavior in a Rodent Model of Traumatic Brain Injury. Brain Res. 1643, 140–151. 10.1016/j.brainres.2016.04.063 27131989 PMC5578618

[B59] Loh K. W. Z. Liang M. C. Soong T. W. Hu Z. (2020). Regulation of Cardiovascular Calcium Channel Activity by post-translational Modifications or Interacting Proteins. Pflugers Arch. 472 (6), 653–667. 10.1007/s00424-020-02398-x 32435990

[B60] Lu C. T. Zhao Y. Z. Wong H. L. Cai J. Peng L. Tian X. Q. (2014). Current Approaches to Enhance CNS Delivery of Drugs across the Brain Barriers. Int. J. Nanomedicine 9, 2241–2257. 10.2147/IJN.S61288 24872687 PMC4026551

[B61] Lucke-Wold B. P. Turner R. C. Logsdon A. F. Bailes J. E. Huber J. D. Rosen C. L. (2014). Linking Traumatic Brain Injury to Chronic Traumatic Encephalopathy: Identification of Potential Mechanisms Leading to Neurofibrillary Tangle Development. J. Neurotrauma 31 (13), 1129–1138. 10.1089/neu.2013.3303 24499307 PMC4089022

[B62] Lucke-Wold B. P. Turner R. C. Logsdon A. F. Nguyen L. Bailes J. E. Lee J. M. (2016). Endoplasmic Reticulum Stress Implicated in Chronic Traumatic Encephalopathy. J. Neurosurg. 124 (3), 687–702. 10.3171/2015.3.JNS141802 26381255 PMC12767288

[B63] Lucke-Wold B. P. Turner R. C. Logsdon A. F. Simpkins J. W. Alkon D. L. Smith K. E. (2015). Common Mechanisms of Alzheimer's Disease and Ischemic Stroke: the Role of Protein Kinase C in the Progression of Age-Related Neurodegeneration. J. Alzheimers Dis. 43 (3), 711–724. 10.3233/JAD-141422 25114088 PMC4446718

[B64] Malenka R. C. Bear M. F. (2004). LTP and LTD: an Embarrassment of Riches. Neuron 44 (1), 5–21. 10.1016/j.neuron.2004.09.012 15450156

[B65] Marais E. Klugbauer N. Hofmann F. (2001). Calcium Channel Alpha(2)delta Subunits-Structure and Gabapentin Binding. Mol. Pharmacol. 59 (5), 1243–1248. 10.1124/mol.59.5.1243 11306709

[B66] Martínez M. Hernández A. I. Hernanz A. (2001). Increased cAMP Immunostaining in Cerebral Vessels in Alzheimer's Disease. Brain Res. 922 (1), 148–152. 10.1016/s0006-8993(01)03009-8 11730714

[B67] Matsuda H. Araki N. Kuji I. Ohkubo T. Imabayashi E. Shimazu K. (2008). Effect of Nilvadipine on Regional Cerebral Blood Flow in a Patient with Early Alzheimer Disease. Clin. Nucl. Med. 33 (1), 34–35. 10.1097/RLU.0b013e31815c4ff0 18097255

[B68] McFarthing K. Simuni T. (2019). Clinical Trial Highlights: Phase III Study in Spotlight. J. Parkinsons Dis. 9 (1), 3–4. 10.3233/JPD-190002 30741695

[B69] Mikami A. Imoto K. Tanabe T. Niidome T. Mori Y. Takeshima H. (1989). Primary Structure and Functional Expression of the Cardiac Dihydropyridine-Sensitive Calcium Channel. Nature 340 (6230), 230–233. 10.1038/340230a0 2474130

[B70] Moon A. L. Haan N. Wilkinson L. S. Thomas K. L. Hall J. (2018). CACNA1C: Association with Psychiatric Disorders, Behavior, and Neurogenesis. Schizophr Bull. 44 (5), 958–965. 10.1093/schbul/sby096 29982775 PMC6101623

[B71] Moosmang S. Haider N. Klugbauer N. Adelsberger H. Langwieser N. Müller J. (2005). Role of Hippocampal Cav1.2 Ca2+ Channels in NMDA Receptor-independent Synaptic Plasticity and Spatial Memory. J. Neurosci. 25 (43), 9883–9892. 10.1523/JNEUROSCI.1531-05.2005 16251435 PMC6725564

[B72] Mullapudi A. Gudala K. Boya C. S. Bansal D. (2016). Risk of Parkinson's Disease in the Users of Antihypertensive Agents: An Evidence from the Meta-Analysis of Observational Studies. J. Neurodegener Dis. 2016, 5780809. 10.1155/2016/5780809 27516917 PMC4969534

[B73] Muralidharan P. Cserne Szappanos H. Ingley E. Hool L. (2016). Evidence for Redox Sensing by a Human Cardiac Calcium Channel. Sci. Rep. 6, 19067. 10.1038/srep19067 26750869 PMC4707475

[B74] Murphy J. G. Sanderson J. L. Gorski J. A. Scott J. D. Catterall W. A. Sather W. A. (2014). AKAP-anchored PKA Maintains Neuronal L-type Calcium Channel Activity and NFAT Transcriptional Signaling. Cell Rep 7 (5), 1577–1588. 10.1016/j.celrep.2014.04.027 24835999 PMC4136445

[B75] Nanou E. Catterall W. A. (2018). Calcium Channels, Synaptic Plasticity, and Neuropsychiatric Disease. Neuron 98 (3), 466–481. 10.1016/j.neuron.2018.03.017 29723500

[B76] Navakkode S. Liu C. Soong T. W. (2018). Altered Function of Neuronal L-type Calcium Channels in Ageing and Neuroinflammation: Implications in Age-Related Synaptic Dysfunction and Cognitive Decline. Ageing Res. Rev. 42, 86–99. 10.1016/j.arr.2018.01.001 29339150

[B77] Núñez-Santana F. L. Oh M. M. Antion M. D. Lee A. Hell J. W. Disterhoft J. F. (2014). Surface L-type Ca2+ Channel Expression Levels Are Increased in Aged hippocampus. Aging Cell 13 (1), 111–120. 10.1111/acel.12157 24033980 PMC3947046

[B78] Nystoriak M. A. Nieves-Cintrón M. Patriarchi T. Buonarati O. R. Prada M. P. Morotti S. (2017). Ser1928 Phosphorylation by PKA Stimulates the L-type Ca2+ Channel CaV1.2 and Vasoconstriction during Acute Hyperglycemia and Diabetes. Sci. Signal. 10 (463), eaaf9647. 10.1126/scisignal.aaf9647 28119464 PMC5297430

[B79] Obeso J. A. Stamelou M. Goetz C. G. Poewe W. Lang A. E. Weintraub D. (2017). Past, Present, and Future of Parkinson's Disease: A Special Essay on the 200th Anniversary of the Shaking Palsy. Mov Disord. 32 (9), 1264–1310. 10.1002/mds.27115 28887905 PMC5685546

[B80] Oertel W. Schulz J. B. (2016). Current and Experimental Treatments of Parkinson Disease: A Guide for Neuroscientists. J. Neurochem. 139 (Suppl. 1), 325–337. 10.1111/jnc.13750 27577098

[B81] Pankonien I. Otto A. Dascal N. Morano I. Haase H. (2012). Ahnak1 Interaction Is Affected by Phosphorylation of Ser-296 on Cavβ₂. Biochem. Biophys. Res. Commun. 421 (2), 184–189. 10.1016/j.bbrc.2012.03.132 22497893

[B82] Paris D. Bachmeier C. Patel N. Quadros A. Volmar C.-H. Laporte V. (2011). Selective Antihypertensive Dihydropyridines Lower Aβ Accumulation by Targeting Both the Production and the Clearance of Aβ across the Blood-Brain Barrier. Mol. Med. 17 (3-4), 149–162. 10.2119/molmed.2010.00180 21170472 PMC3060987

[B83] Park H. J. Min S. H. Won Y. J. Lee J. H. (2015). Asn-Linked Glycosylation Contributes to Surface Expression and Voltage-dependent Gating of Cav1.2 Ca²⁺ Channel. J. Microbiol. Biotechnol. 25 (8), 1371–1379. 10.4014/jmb.1501.01066 25824433

[B84] Parkinson Study Group (2013). Phase II Safety, Tolerability, and Dose Selection Study of Isradipine as a Potential Disease-Modifying Intervention in Early Parkinson's Disease (STEADY-PD). Mov Disord. 28 (13), 1823–1831. 10.1002/mds.25639 24123224

[B85] Parkinson Study Group STEADY-PD III Investigators (2020). Isradipine versus Placebo in Early Parkinson Disease: A Randomized Trial. Ann. Intern. Med. 172 (9), 591–598. 10.7326/M19-2534 32227247 PMC7465126

[B86] Pasternak B. Svanström H. Nielsen N. M. Fugger L. Melbye M. Hviid A. (2012). Use of Calcium Channel Blockers and Parkinson's Disease. Am. J. Epidemiol. 175 (7), 627–635. 10.1093/aje/kwr362 22387374

[B87] Patel M. M. Goyal B. R. Bhadada S. V. Bhatt J. S. Amin A. F. (2009). Getting into the Brain: Approaches to Enhance Brain Drug Delivery. CNS Drugs 23 (1), 35–58. 10.2165/0023210-200923010-00003 19062774

[B88] Perez-Reyes E. Castellano A. Kim H. S. Bertrand P. Baggstrom E. Lacerda A. E. (1992). Cloning and Expression of a Cardiac/brain Beta Subunit of the L-type Calcium Channel. J. Biol. Chem. 267 (3), 1792–1797. 10.1016/s0021-9258(18)46015-2 1370480

[B89] Perovic M. Tesic V. Mladenovic Djordjevic A. Smiljanic K. Loncarevic-Vasiljkovic N. Ruzdijic S. (2013). BDNF Transcripts, proBDNF and proNGF, in the Cortex and hippocampus throughout the Life Span of the Rat. Age (Dordr) 35 (6), 2057–2070. 10.1007/s11357-012-9495-6 23255148 PMC3824987

[B90] Popovic D. Vucic D. Dikic I. (2014). Ubiquitination in Disease Pathogenesis and Treatment. Nat. Med. 20 (11), 1242–1253. 10.1038/nm.3739 25375928

[B91] Qian H. Patriarchi T. Price J. L. Matt L. Lee B. Nieves-Cintrón M. (2017). Phosphorylation of Ser1928 Mediates the Enhanced Activity of the L-type Ca2+ Channel Cav1.2 by the β2-adrenergic Receptor in Neurons. Sci. Signal. 10 (463). 10.1126/scisignal.aaf9659 PMC5310946 28119465

[B92] Ritz B. Rhodes S. L. Qian L. Schernhammer E. Olsen J. H. Friis S. (2010). L-Type Calcium Channel Blockers and Parkinson Disease in Denmark. Ann. Neurol. 67 (5), 600–606. 10.1002/ana.21937 20437557 PMC2917467

[B93] Sanders O. Rajagopal L. (2020). Phosphodiesterase Inhibitors for Alzheimer's Disease: A Systematic Review of Clinical Trials and Epidemiology with a Mechanistic Rationale. J. Alzheimers Dis. Rep. 4 (1), 185–215. 10.3233/ADR-200191 32715279 PMC7369141

[B94] Schulz J. B. Hausmann L. Hardy J. (2016). 199 Years of Parkinson Disease - what Have We Learned and what Is the Path to the Future? J. Neurochem. 139 (Suppl. 1), 3–7. 10.1111/jnc.13733 27581372

[B95] Sculptoreanu A. Rotman E. Takahashi M. Scheuer T. Catterall W. A. (1993). Voltage-dependent Potentiation of the Activity of Cardiac L-type Calcium Channel Alpha 1 Subunits Due to Phosphorylation by cAMP-dependent Protein Kinase. Proc. Natl. Acad. Sci. U S A. 90 (21), 10135–10139. 10.1073/pnas.90.21.10135 7694283 PMC47728

[B96] Singh A. Verma P. Balaji G. Samantaray S. Mohanakumar K. P. (2016). Nimodipine, an L-type Calcium Channel Blocker Attenuates Mitochondrial Dysfunctions to Protect against 1-Methyl-4-Phenyl-1,2,3,6-Tetrahydropyridine-Induced Parkinsonism in Mice. Neurochem. Int. 99, 221–232. 10.1016/j.neuint.2016.07.003 27395789

[B97] Splawski I. Timothy K. W. Sharpe L. M. Decher N. Kumar P. Bloise R. (2004). Ca(V)1.2 Calcium Channel Dysfunction Causes a Multisystem Disorder Including Arrhythmia and Autism. Cell 119 (1), 19–31. 10.1016/j.cell.2004.09.011 15454078

[B98] Striessnig J. Pinggera A. Kaur G. Bock G. Tuluc P. (2014). L-type Ca2+ Channels in Heart and Brain. Wiley Interdiscip. Rev. Membr. Transp Signal. 3 (2), 15–38. 10.1002/wmts.102 24683526 PMC3968275

[B99] Surguchov A. (2020). Caveolin: A New Link between Diabetes and AD. Cell Mol Neurobiol 40 (7), 1059–1066. 10.1007/s10571-020-00796-4 31974905 PMC11448860

[B100] Swatek K. N. Komander D. (2016). Ubiquitin Modifications. Cell Res 26 (4), 399–422. 10.1038/cr.2016.39 27012465 PMC4822133

[B101] Tang H. Viola H. M. Filipovska A. Hool L. C. (2011). Ca(v)1.2 Calcium Channel Is Glutathionylated during Oxidative Stress in guinea Pig and Ischemic Human Heart. Free Radic. Biol. Med. 51 (8), 1501–1511. 10.1016/j.freeradbiomed.2011.07.005 21810465

[B102] Tétreault M. P. Bourdin B. Briot J. Segura E. Lesage S. Fiset C. (2016). Identification of Glycosylation Sites Essential for Surface Expression of the CaVα2δ1 Subunit and Modulation of the Cardiac CaV1.2 Channel Activity. J. Biol. Chem. 291 (9), 4826–4843. 10.1074/jbc.M115.692178 26742847 PMC4813503

[B103] Uchida S. Yamada S. Nagai K. Deguchi Y. Kimura R. (1997). Brain Pharmacokinetics and *In Vivo* Receptor Binding of 1,4-dihydropyridine Calcium Channel Antagonists. Life Sci. 61 (21), 2083–2090. 10.1016/s0024-3205(97)00881-3 9395249

[B104] Venuto C. S. Yang L. Javidnia M. Oakes D. James Surmeier D. Simuni T. (2021). Isradipine Plasma Pharmacokinetics and Exposure-Response in Early Parkinson's Disease. Ann. Clin. Transl Neurol. 8 (3), 603–612. 10.1002/acn3.51300 33460320 PMC7951102

[B105] Villacrés C. Tayi V. S. Lattová E. Perreault H. Butler M. (2015). Low Glucose Depletes Glycan Precursors, Reduces Site Occupancy and Galactosylation of a Monoclonal Antibody in CHO Cell Culture. Biotechnol. J. 10 (7), 1051–1066. 10.1002/biot.201400662 26058832

[B106] Walker D. M. Nestler E. J. (2018). Neuroepigenetics and Addiction. Handb Clin. Neurol. 148, 747–765. 10.1016/B978-0-444-64076-5.00048-X 29478612 PMC5868351

[B107] Wang D. Papp A. C. Binkley P. F. Johnson J. A. Sadée W. (2006). Highly Variable mRNA Expression and Splicing of L-type Voltage-dependent Calcium Channel Alpha Subunit 1C in Human Heart Tissues. Pharmacogenet Genomics 16 (10), 735–745. 10.1097/01.fpc.0000230119.34205.8a 17001293 PMC2688811

[B108] Wang Q. M. Xu Y. Y. Liu S. Ma Z. G. (2017). Isradipine Attenuates MPTP-Induced Dopamine Neuron Degeneration by Inhibiting Up-Regulation of L-type Calcium Channels and Iron Accumulation in the Substantia Nigra of Mice. Oncotarget 8 (29), 47284–47295. 10.18632/oncotarget.17618 28521299 PMC5564564

[B109] Wang R. Ma Z. Wang J. Xie J. (2012). L-type Cav1.2 Calcium Channel Is Involved in 6-Hydroxydopamine-Induced Neurotoxicity in Rats. Neurotox Res. 21 (3), 266–270. 10.1007/s12640-011-9271-x 21901331

[B110] Weiss S. Keren-Raifman T. Oz S. Ben Mocha A. Haase H. Dascal N. (2012). Modulation of Distinct Isoforms of L-type Calcium Channels by G(q)-coupled Receptors in Xenopus Oocytes: Antagonistic Effects of Gβγ and Protein Kinase C. Channels (Austin) 6 (6), 426–437. 10.4161/chan.22016 22990911 PMC3536727

[B111] Whitcomb V. Wauson E. Christian D. Clayton S. Giles J. Tran Q. K. (2020). Regulation of Beta Adrenoceptor-Mediated Myocardial Contraction and Calcium Dynamics by the G Protein-Coupled Estrogen Receptor 1. Biochem. Pharmacol. 171, 113727. 10.1016/j.bcp.2019.113727 31759979 PMC6952073

[B112] Xu H. Ginsburg K. S. Hall D. D. Zimmermann M. Stein I. S. Zhang M. (2010). Targeting of Protein Phosphatases PP2A and PP2B to the C-Terminus of the L-type Calcium Channel Ca v1.2. Biochemistry 49 (48), 10298–10307. 10.1021/bi101018c 21053940 PMC3075818

[B113] Xu W. Lipscombe D. (2001). Neuronal Ca(V)1.3alpha(1) L-type Channels Activate at Relatively Hyperpolarized Membrane Potentials and Are Incompletely Inhibited by Dihydropyridines. J. Neurosci. 21 (16), 5944–5951. 10.1523/jneurosci.21-16-05944.2001 11487617 PMC6763157

[B114] Yang L. Dai D. F. Yuan C. Westenbroek R. E. Yu H. West N. (2016). Loss of β-adrenergic-stimulated Phosphorylation of CaV1.2 Channels on Ser1700 Leads to Heart Failure. Proc. Natl. Acad. Sci. U S A. 113 (49), E7976–E7985. 10.1073/pnas.1617116113 27864509 PMC5150375

[B115] Yang L. Liu G. Zakharov S. I. Bellinger A. M. Mongillo M. Marx S. O. (2007). Protein Kinase G Phosphorylates Cav1.2 Alpha1c and Beta2 Subunits. Circ. Res. 101 (5), 465–474. 10.1161/CIRCRESAHA.107.156976 17626895

[B116] Yang L. Liu G. Zakharov S. I. Morrow J. P. Rybin V. O. Steinberg S. F. (2005). Ser1928 Is a Common Site for Cav1.2 Phosphorylation by Protein Kinase C Isoforms. J. Biol. Chem. 280 (1), 207–214. 10.1074/jbc.M410509200 15509562

[B117] Yasar S. Corrada M. Brookmeyer R. Kawas C. (2005). Calcium Channel Blockers and Risk of AD: the Baltimore Longitudinal Study of Aging. Neurobiol. Aging 26 (2), 157–163. 10.1016/j.neurobiolaging.2004.03.009 15582745

[B118] Zamponi G. W. Striessnig J. Koschak A. Dolphin A. C. (2015). The Physiology, Pathology, and Pharmacology of Voltage-Gated Calcium Channels and Their Future Therapeutic Potential. Pharmacol. Rev. 67 (4), 821–870. 10.1124/pr.114.009654 26362469 PMC4630564

[B119] Zhang Y. Q. Sarge K. D. (2008). Sumoylation of Amyloid Precursor Protein Negatively Regulates Abeta Aggregate Levels. Biochem. Biophys. Res. Commun. 374 (4), 673–678. 10.1016/j.bbrc.2008.07.109 18675254 PMC2596940

